# Are we there yet? Pushing the boundaries of endonasal surgery into the brainstem

**DOI:** 10.3171/2020.4.FocusVid.2020

**Published:** 2020-04-01

**Authors:** Aaron A. Cohen-Gadol

**Affiliations:** Associate Editor-in-Chief, *Neurosurgical Focus: Video*;1 Department of Neurosurgery, Indiana University, Indianapolis; and; 2 *The Neurosurgical Atlas*, Indianapolis, Indiana

Innovation distinguishes between a leader and a follower

This quote by Steve Jobs has defined the most admired surgeons in our discipline. Innovation fueled by surgical intelligence can expand the reach and performance of surgery beyond what was once considered possible. Endonasal surgery has shepherded skull base surgery into an era in which some of the radical open skull base exposures have been replaced with minimally invasive endonasal corridors, resulting in the concept of operative space being replaced by operative angle.

The video titled the “Endoscopic Transnasal Resection of the CP Angle Schwannoma” by Kiyofuji and colleagues in this issue of *Neurosurgical Focus: Video* demonstrates an effort to push the boundaries of endonasal surgery for resection of an intradural lateral brainstem tumor. Although the retromastoid approach would have provided reasonable exposure of this lesion and potentially more aggressive resection while placing the VI nerve at less risk, the authors chose the endonasal route and were able to achieve a good result. In this video, the limitations of microsurgical instrumentation in carrying out intradural microsurgery within lateral trajectories in this operative space are evident. The angles of freedom become progressively more restrictive and challenge the microsurgical prowess of the surgeon to achieve maximal resection.

Pushing the boundaries of minimally invasive surgical approaches at the expense of increased technical difficulty and its yield of resection frequently haunts us. Our ability to resect brainstem lesions via endonasal approaches is at its early stages and requires further evolution of endoscopic microinstrumentation and development of objective selection criteria. Ultimately, the operation is not only about how to get there but also, more importantly, about what to do when you are there. I am thankful to Dr. Kiyofuji and colleagues for their thought-provoking video.

